# Comparable type I interferon score determination from PAXgene and Tempus whole blood RNA collection and isolation systems

**DOI:** 10.1186/s13104-019-4562-z

**Published:** 2019-08-15

**Authors:** Lovro Lamot, Iwona Niemietz, Kelly L. Brown

**Affiliations:** 1 Division of Rheumatology, Department of Pediatrics, Faculty of Medicine, The University of British Columbia, Vancouver, BC Canada; 2 BC Children’s Hospital Research Institute, Rm A4-145, 950 West 28th Ave, Vancouver, BC V5Z 4H4 Canada; 3 Department of Microbiology & Immunology, Faculty of Science, The University of British Columbia, Vancouver, BC Canada; 4 Centre for Blood Research, Faculty of Medicine, The University of British Columbia, Vancouver, BC Canada

**Keywords:** Interferon score, Interferon stimulated genes, PAXgene, Tempus, RNA

## Abstract

**Objective:**

Type I interferons (IFN) have important roles in many immune-mediated inflammatory diseases (IMIDs) and are a relatively new therapeutic target. Direct detection of type I IFNs has proved challenging, thus their presence is often inferred from the expression of interferon-stimulated genes (ISGs) and calculation of an interferon score (IS). The objective of this research was to determine if the expression of six common ISGs and subsequent IS were comparable when RNA was derived from the Tempus and PAXgene whole blood RNA collection systems.

**Results:**

Whole blood was obtained from ten healthy adults, incubated ex vivo in the absence and presence of recombinant human IFNα then divided into PAXgene and Tempus tubes. Despite reports of tube-specific patterns of gene expression, quantitative PCR (qPCR) analysis revealed no significant differences between PAXgene and Tempus tubes in either the homeostatic or IFNα-induced expression of six ISGs (IFI27, IFI44L, IFIT1, ISG15, RSAD2, SIGLEC1). Overall there was a strong correlation in the IS between unstimulated (r = 0.92, p = 0.0005) and IFNα-stimulated (r = 0.71, p = 0.0268) samples derived from the PAXgene and Tempus tubes.

**Electronic supplementary material:**

The online version of this article (10.1186/s13104-019-4562-z) contains supplementary material, which is available to authorized users.

## Introduction

Type I interferons (IFN) are a class of inducible and protective cytokines with a breadth of immune-modulatory functions that are important for defence against various pathogens [1]. Persistent type I IFN activity however can have detrimental effects that have been associated with a growing number of immune-mediated inflammatory disorders (IMIDs): the most obvious of which are the type I interferonopathies, but also include other autoinflammatory and rheumatic diseases such as systemic lupus erythematosus (SLE), dermatomyositis (DM), rheumatoid arthritis (RA) and systemic juvenile idiopathic arthritis (sJIA) [2–4]. As such, type I IFNs are one of the newest therapeutic targets and several “anti-interferon (anti-IFN)” treatments are currently in use or in the final stages of clinical trials (for e.g., JAK inhibitors, reverse transcriptase inhibitors and monoclonal antibodies) [5].

For a variety of reasons, the direct detection of type I interferons in biologic samples has proved challenging. Thus, indirect methods are often used to infer the presence of type I IFN [6]. Most often this involves quantification of the relative expression of interferon-stimulated genes (ISGs) which are then used to calculate an interferon score (IS) [7]. The expression of six ISGs (IFI27, IFI44L, IFIT1, ISIG15, RSAD2, SIGLEC1) have been measured and used to calculate an IS in the majority of studies involving patients with IMIDs: these ISGs have the greatest differential expression between healthy individuals and patients with Aicardi–Goutières syndrome (AGS), the first defined type I interferonopathy [8, 9].

While assessment of type I IFN has clinical utility for diagnosis and disease/treatment monitoring, there exists no consensus approach or standardized protocols for accurate and timely type I IFN quantification. A recent study showed that qPCR and Nanostring technology have similar sensitivity and reproducibility for IS determination [10]. The impact of different methods to obtain whole blood RNA however have not been considered. PAXgene and Tempus are two commonly used commercial sampling systems for the isolation of high-quality RNA from blood, and while they have the same purpose, reproducible differences in gene expression between the systems have been reported; these differences could limit the use, or at least comparative analysis, of samples from existing biobanks and/or laboratories employing different isolation techniques [11–18]. Specifically, differences in transcript abundance of two ISGs, IFI44L and IFIT1, have been noted between the Tempus and PAXgene sampling systems [18]. The aim of this study was to compare the expression of six common ISGs and the corresponding IS in RNA isolated from PAXgene versus Tempus whole blood collection systems.

## Main text

### Methods

#### Blood collection, RNA isolation and cDNA synthesis

After obtaining informed consent (see “Ethics approval and consent to participate” section), 15 ml of whole blood was collected from ten healthy individuals over 19 years of age (median age 25.5 years, range 19–38 years, 4 male, 6 female) in sodium heparin tubes (Becton–Dickinson), divided and incubated without (unstimulated, 6 ml) and with (stimulated, 6 ml) recombinant human interferon alpha 2b (Novus Biologicals, rhIFNα, 12 IU/6 ml blood, 4 h, 37 °C, 5% CO_2_). Stimulated and unstimulated blood was transferred to both PAXgene (2.5 ml blood, PreAnalytiX, Becton–Dickinson) and Tempus (3 ml blood, Applied Biosystems) tubes, incubated 24 h at room temperature and stored at − 80 °C. Within 2–4 weeks of storage, RNA was isolated according to the respective manufacturer’s protocol with the addition of RNase-free DNAse during the isolation procedure (Qiagen for Tempus, PreanalytiX for PAXgene, 40.9 Kunitz units/sample, 15 min) and inclusion of RNaseOUT Recombinant Ribonuclease Inhibitor (Invitrogen, 40 units/sample) in each eluted RNA sample. A NanoDrop ND-1000 spectrophotometer (Thermo Fisher Scientific) was used to measure RNA concentration and integrity. The first strand of cDNA was reverse transcribed from ~ 500 ng of RNA using the qScript cDNA synthesis kit (QuantaBio) according to manufacturer’s instructions and resultant cDNA was stored at − 20 °C.

#### PCR and interferon score calculation

The expression of six ISGs and two housekeeping genes (Table 1) typically used for Interferon score determination were measured by qPCR using TaqMan Fast Advanced Mastermix and Assays (Applied Biosystems) as per manufacturer’s protocol [7]. Assays were run in triplicate on fast optical 96-well plates (Applied Biosystems) using a QuantStudio 6 Real-Time PCR instrument (Thermo Fisher Scientific). Reaction volumes contained 1 μl of reverse transcribed cDNA in a total reaction volume of 10 μl. PCR conditions were as follows: 2 min at 50 °C, 20 s at 95 °C, 40 cycles of 1 s at 95 °C and 20 s at 60 °C. For each ISG, expression was normalized against the geometric mean of two housekeeping genes (HPRT1 and 18S rRNA) and calculated using the formula 2^−ΔCt^ [19, 20]. Relative expression was reported as the normalized expression of each ISG divided by the median of normalized expression of the same ISG in unstimulated samples derived from the PAXgene or Tempus tubes, respectively. The median relative expression of all six ISGs was used to calculate the IFN score for each sample [8].

**Table 1:** Genes used for quantification by qPCR and interferon score calculation

Gene symbol and name	TaqMan assay ID
SELECTED ISGs	
IFI27: interferon alpha inducible protein 27	Hs01086370_m1
IFI44L: interferon induced protein 44 like	Hs00199115_m1
IFIT1: interferon induced protein with tetratricopeptide repeats 1	Hs00356631_g1
ISG15: interferon-stimulated gene 15	Hs00192713_m1
RSAD2: radical *S*-adenosyl methionine domain containing 2	Hs01057264_m1
SIGLEC1: sialic acid binding Ig like lectin 1	Hs00988063_m1
Housekeeping genes	
18S rRNA: 18 S ribosomal RNA	Hs999999001_s1
HPRT1: hypoxanthine phosphoribosyltransferase 1	Hs03929096_g1

#### Statistical analysis

Statistical analysis was performed using GraphPad Prism 7 for Mac OS X software (GraphPad software, version 7.0c). Wilcoxon matched-pairs signed rank test was used to compare expression of the six ISGs between stimulated and unstimulated samples derived from PAXgene or Tempus tubes. Spearman correlation was used to determine the correlation of interferon scores derived from either PAXgene or Tempus tubes.

### Results

#### PAXgene and Tempus tubes yield comparable expression of interferon stimulated genes

**Fig. 1:**
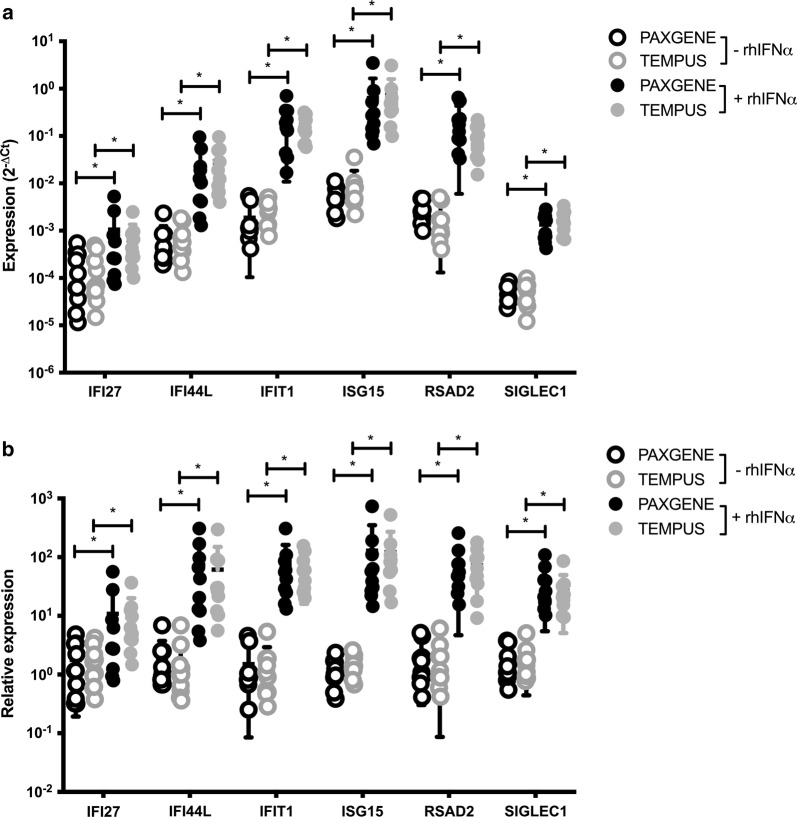
Expression of Interferon-stimulated genes in whole blood RNA derived from PAXgene and Tempus tubes. Data points represent the **a** mean expression ± standard deviation (SD) and **b** mean relative expression ± SD (y-axis) in each of ten healthy individuals (n = 10) of interferon-stimulated genes (x-axis) following 4 h incubation ex vivo in the absence (open circles) and presence (solid circles) of rhIFNα followed by RNA collection and processing in Tempus (grey) and PAXgene (black) tubes. *Indicates p < 0.005

In this study we first evaluated the baseline (unstimulated) whole blood expression of each of the six ISGs of interest (IFI27, IFI44L, IFIT1, ISIG15, RSAD2, SIGLEC1) in samples derived from the Tempus and PAXgene tubes. Results obtained by qPCR demonstrated that the baseline relative expression of each ISG in our cohort was similar to the relative expression reported for these genes in other cohorts of healthy volunteers [10]. Moreover, our data revealed no statistically significant difference in baseline expression for any of the six ISGs between the RNA collection tubes (Fig. 1a, open circles, n = 10 donors).

Next, we measured the expression of each ISG in whole blood stimulated ex vivo with recombinant human (rh) IFNα to mimic elevated type I IFN in vivo. As expected, a statistically significant increase of expression (compared to the baseline expression in each individual) was observed for every ISG in rhIFNα-stimulated (Fig. 1a, solid circles, n = 10 donors). Mean relative expression was upregulated over baseline for all genes in both PAXgene and Tempus tubes (Fig. 1b, solid circles, normalized to baseline (open circles), n = 10 donors).

Although the magnitude of induced expression for each gene, as may be expected, varied between donors, results from the PAXgene and Tempus tubes were similar for each rhIFNα-induced ISG regardless of the magnitude of expression (Additional file 1: Figure S1); for e.g., IFI27 and ISG15, respectively, had the weakest and strongest induction yet the mean relative expression in PAXgene versus Tempus tubes was 10.9 versus 9.6 for IFI27 and 129.4 versus 121.5 for ISG15. Thus, our results show no significant difference across ten healthy donors in baseline or rhIFNα-induced expression of six ISGs in whole blood RNA derived from PAXgene and Tempus tubes.

#### Interferon score calculated from PAXgene and Tempus tubes are highly correlated

**Fig. 2:**
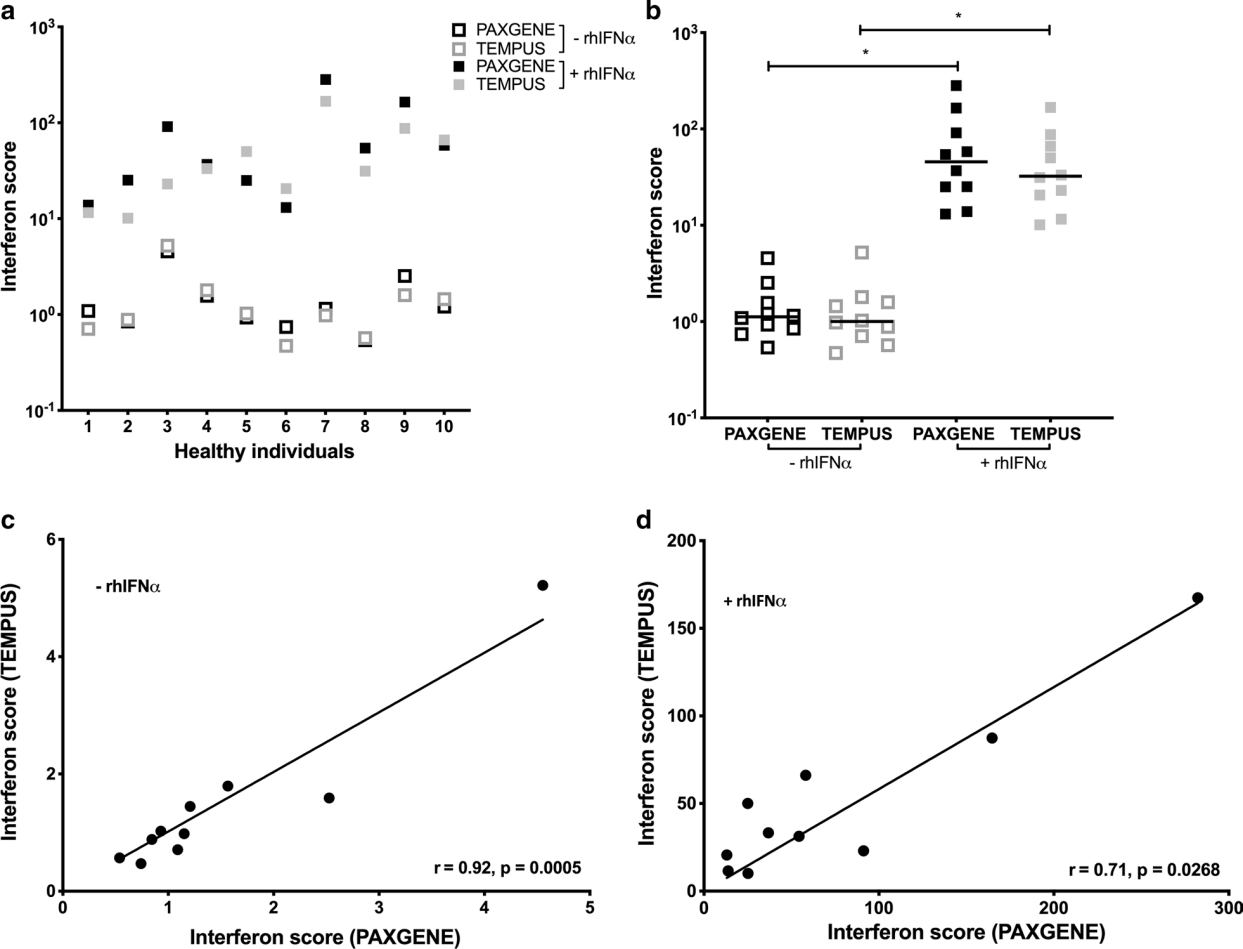
Interferon score derived from PAXgene and Tempus tubes. **a**, **b**. Interferon score (y-axis) calculated for ten healthy individuals (x-axis; **a**) following 4 h ex vivo incubation of whole blood in the absence (open squares) and presence (solid squares) of rhIFNα and subsequent collection in PAXgene (black) and Tempus (grey) tubes (x-axis; **b**). Horizontal lines represent the median interferon score (n = 10; **b**). *Indicates p < 0.005. **c**, **d** Spearman correlation (r) and p-value of the interferon score in samples (n = 10) isolated from PAXgene (y-axis) and Tempus tubes (x-axis) without (**c**) and with (**d**) rhIFNα stimulation

Next we calculated the interferon score (IS) for each individual prior to and following rhIFNα stimulation (Fig. 2a, b). For unstimulated samples (Fig. 2a, b open boxes), the median IS (n = 10) derived from the relative ISG expression in PAXgene and Tempus tubes, respectively, was 1.1 (range 0.5–4.6) and 1.0 (range 0.5–5.2). These scores were strongly correlated between PAXgene and Tempus tubes (Fig. 2c, r = 0.92, p = 0.0005).

From rhIFNα-stimulated samples (Fig. 2a, b solid boxes), the median IS (n = 10) derived from the relative ISG expression in PAXgene and Tempus tubes, respectively, was 45.6 and 32.3. Calculated scores, like the ISG expression from which they were derived, varied between individuals (range 13.1–282.3 for PAXgene and 10.1–167.4 for Tempus). Despite this, there was a strong correlation of the IS between PAXgene and Tempus tubes for individual rhIFNα-stimulated samples (r = 0.71, p = 0.0268) (Fig. 2d).

The threshold for a positive interferon score was defined as the mean value of the interferon score + 2 standard deviations (SD) in unstimulated samples (n = 10) [10]. Using this criteria, we discovered that the IS calculated from both Tempus and PAXgene tubes was positive for two individuals (Fig. 2a, individual #3 and #9). After exclusion of data from these individuals, it was determined that an interferon score exceeding 1.72 or 2.25 (n = 8) was considered positive for samples derived, respectively from PAXgene and Tempus tubes.

### Discussion

Despite reported differences in gene expression patterns associated with blood samples collected in PAXgene and Tempus tubes—including interferon stimulated genes IFI44L and IFIT1 [18]—our results demonstrate that the expression of six ISGs (IFI27, IFI44L, IFIT1, ISG15, RSAD2 and SIGLEC) and derived interferon scores are comparable in whole blood RNA obtained with PAXgene and Tempus isolation systems.

In our experimental system, ex vivo stimulation for 4 h of whole blood from healthy donors with rhIFNα was sufficient to induce relatively high levels of expression of all six ISGs tested. The magnitude of relative expression, as may be expected, varied between individuals, but was comparable between Tempus and PAXgene tubes for each ISG in every individual. In contrast to other reports, we did not find consistently higher Ct values in samples isolated from PAXgene tubes (implying the lower abundance of some transcripts associated with PAXgene tubes in those studies) [15, 17].

While typically the expression of IFI27 is one of the highest amongst the ISGs in patients with type I interferon associated diseases, the ex vivo induction of IFI27 in whole blood from healthy donors was relatively weak compared to the other ISGs, and fivefold lower when compared to patients [10]. In contrast, the relative expression of the other tested ISGs (IFI44L, IFIT1, ISG15, RSAD2 and SIGLEC) was three- to tenfold higher than that reported in patients with monogenic interferonopathy (n = 6), systemic erythematous lupus (n = 26), and dermatomyositis (n = 8) [10]. While detailed analysis of differential gene expression patterns may be important to improve the understanding of type I IFN signaling and interferonopathies, the IS calculation allows for these differences as it considers the median relative expression and central distribution tendency rather than the precise pattern of ISG expression and small differences in Ct values.

A range of positive interferon scores (~ 10–280) was generated within our cohort consistent with the varied magnitude of induced ISG expression. Of importance, our interferon scores encompassed the range of IS reported for patients with type I interferon associated diseases [2, 8, 10, 21]. Across the range, calculated scores were highly correlated between PAXgene and Tempus tubes for individual samples and the entire cohort.

One of the challenges in the calculation of an IS is establishing the threshold for categorizing scores as positive or negative. There is no consensus on an appropriate ‘negative’ control with some studies using samples from single individuals as a baseline for comparison. With either individual measures or pooled reference controls, our results suggest that control samples isolated from either PAXgene and Tempus tubes can be used to establish the threshold for a positive IS and can be applied to the analysis of samples irrespective of collection tube. However, due to normal inter-individual variability in ISG expression and potential for asymptomatic elevated baseline expression of ISGs in healthy individuals (possibly due to a recent viral exposure) our data emphasize the importance of constructing a comparator sample from multiple individuals that have been pre-screened for ISG expression.

While the standardization of methods for type I IFN score determination are still needed, our results suggest that the whole blood expression of each of six ISGs is similar in Tempus and PAXgene RNA collection systems, and health care and research centres can use either PAXgene or Tempus tubes for IFN score determination.

## Limitations

In this study, we compare two whole blood RNA collection systems with respect to the expression of six type I IFN stimulated genes and calculated interferon score. To do this, we used blood from healthy individuals incubated ex vivo in the absence and presence of recombinant IFNα. Our results demonstrate that different ISGs are induced to a different extent compared to that in patients. While it is likely that our findings would remain unchanged, the absence of patient samples in our study is a potential limitation.

## Additional file

**Additional file 1: Figure S1.**  Baseline and rhIFNα induced expression of six interferon stimulated genes in ten healthy individuals following RNA collection and processing in Tempus and PAXgene tubes.

## Data Availability

All data generated or analysed during this study are included in this published article and its additional files.

## Abbreviations

qPCR: quantitative polymerase chain reaction
IFN: interferon
IS: interferon score
ISG: interferon stimulated gene
rhIFNα: recombinant human interferon alpha 2b
AGS: Aicardi–Goutières syndrome
IMID: immune-mediated inflammatory disorders
SLE: systemic lupus erythematosus
DM: dermatomyositis
RA: rheumatoid arthritis
sJIA: systemic juvenile idiopathic arthritis
