# Supplementary material for: Comparable type I interferon score determination from PAXgene and Tempus whole blood RNA collection and isolation systems
Source: BMC Res Notes. 2019 Aug 15;12:511. doi: 10.1186/s13104-019-4562-z (PMC6694656; doi:10.1186/s13104-019-4562-z)

**Figure S1. Baseline and IFNα-induced expression of Interferon-stimulated genes in ten healthy individuals.** Data points represent the (A) expression and (B) relative expression (y-axis) for each of six interferon stimulated genes (IFI27, IFI44L, IFIT1, ISG15, RSAD2, SIGLEC1) in ten otherwise healthy adults (x-axis) following 4 hr incubation ex vivo in the absence (open circles) and presence (solid circles) of rhIFNα followed by RNA collection and processing in Tempus (grey) and PAXgene (black) tubes.


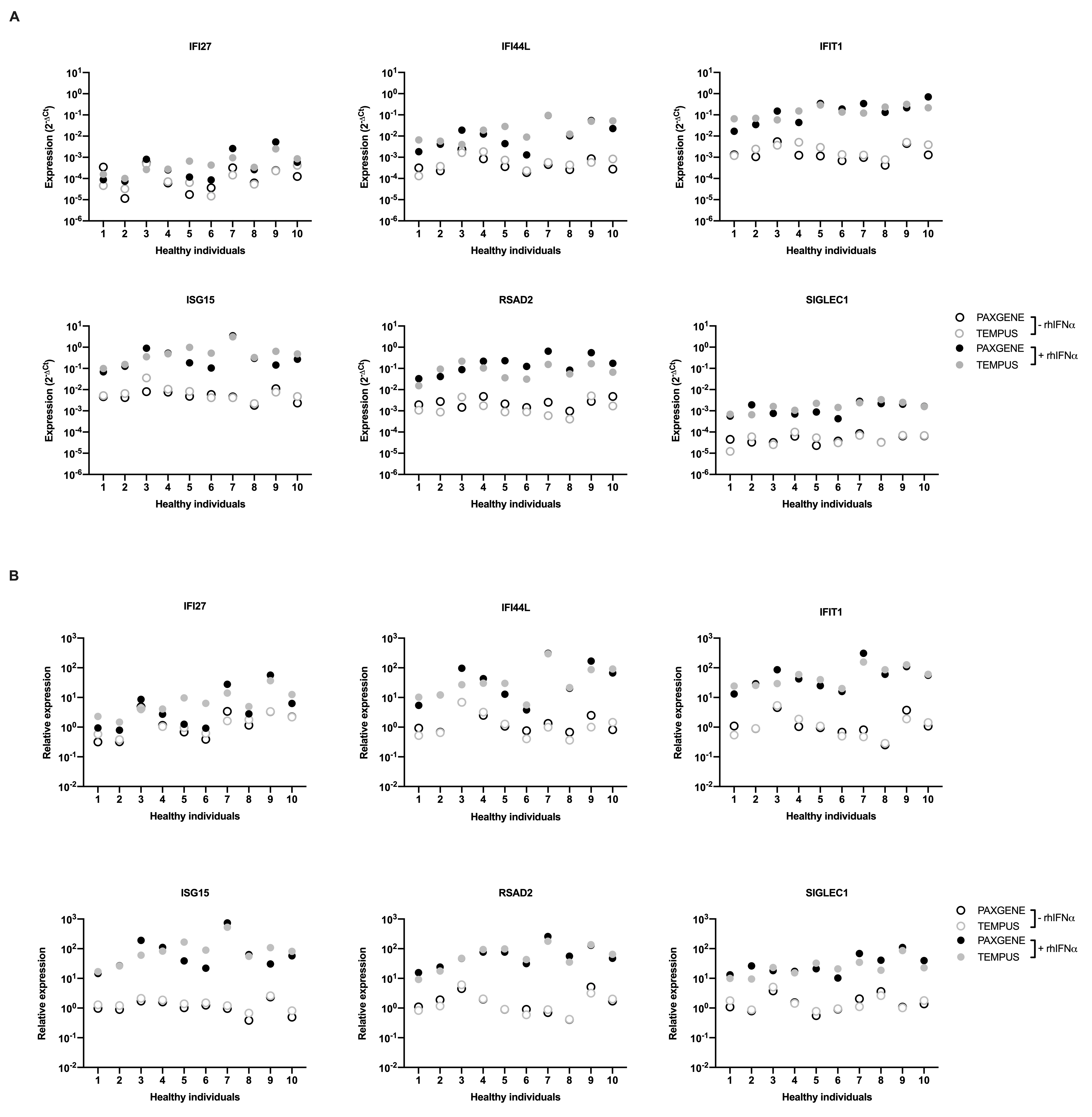

Supplement: Supplementary file 1 — Additional file 1: Figure S1. Baseline and rhIFNα induced expression of six interferon stimulated genes in ten healthy individuals following RNA collection and processing in Tempus and PAXgene tubes. [file 13104_2019_4562_MOESM1_ESM.docx]
